# Prognostic Electrocardiographic Signs in Arrhythmogenic Cardiomyopathy

**DOI:** 10.3390/biology13040265

**Published:** 2024-04-16

**Authors:** Elisabetta Tonet, Francesco Vitali, Veronica Amantea, Giorgia Azzolini, Cristina Balla, Marco Micillo, Davide Lapolla, Luca Canovi, Matteo Bertini

**Affiliations:** Cardiovascular Institute, Azienda Ospedaliero-Universitaria of Ferrara, 44124 Cona, Italy; tonet.elisabetta@gmail.com (E.T.); veronica.amantea@gmail.com (V.A.); azzolini.giorgia@libero.it (G.A.); bllcst@unife.it (C.B.); marco.micillo942@gmail.com (M.M.); davidelapolla@gmail.com (D.L.); lucacanov@gmail.com (L.C.); doc.matber@gmail.com (M.B.)

**Keywords:** arrhythmogenic cardiomyopathy, electrocardiography, prognosis

## Abstract

**Simple Summary:**

The electrocardiogram (ECG) represents one of the main instruments used by cardiologists for patient evaluation and diagnosis formulation. It is used for some types of cardiomyopathy, such as arrhythmogenic cardiomyopathy (ACM). In this setting, particular ECG signs have also been related to prognosis. Our review summarizes the ACM ECG signs that have a demonstrated prognostic role.

**Abstract:**

Arrhythmogenic cardiomyopathy (ACM) is a rare cardiac disease, characterized by the progressive replacement of myocardial tissue with fibrous and fatty deposits. It can involve both the right and left ventricles. It is associated with the development of life-threatening arrhythmias and culminates in sudden cardiac death. Electrocardiography (ECG) has emerged as a pivotal tool, offering diagnostic insights and prognostic information. The specific ECG abnormalities observed in ACM not only contribute to early detection but also hold the key to the prediction of the likelihood of severe complications. The recognition of these nuanced ECG manifestations has become imperative for clinicians as it guides them in the formulation of tailored therapeutic strategies that address both the present symptoms and the potential future risks.

## 1. Introduction

Arrhythmogenic cardiomyopathy (ACM) stands as a rare yet menacing cardiac disorder, characterized by the progressive replacement of myocardial tissue with fibrous and fatty deposits; it primarily affects the right ventricle. The chief concern lies in the potential development of life-threatening arrhythmias, culminating in sudden cardiac death [1].

In the diagnostic landscape of ACM, electrocardiography (ECG) emerges as a pivotal tool, offering not only diagnostic insights but also invaluable prognostic information. The distinct ECG signs associated with ACM serve as early harbingers of the disease’s trajectory, unveiling crucial details about the electrical disturbances resulting from pathological changes in the cardiac tissue [1].

As we delve into the intricacies of ACM, it becomes apparent that ECG serves not merely as a diagnostic aid but also as a prognostic compass. The prognostic importance of ECG findings is widely recognized, as they serve as critical indicators of disease severity and risk stratification. ECG abnormalities are often among the earliest detectable signs of ACM and can provide valuable insights into the underlying myocardial structural and electrical abnormalities, aiding in both diagnosis and prognostication [1].

Specific ECG findings are commonly observed in ACM and are associated with an increased risk of adverse cardiac events, including ventricular arrhythmias and sudden cardiac death. The presence, pattern, and extent of these abnormalities can help clinicians assess the severity of myocardial involvement and the likelihood of future arrhythmic events. Moreover, serial ECG assessments allow disease progression to be monitored [1].

In addition to aiding in risk stratification and disease monitoring, ECG findings also inform therapeutic decisions in ACM. For example, the presence of certain ECG abnormalities may prompt the consideration of implantable cardioverter–defibrillator (ICD) placement for the primary prevention of sudden cardiac death, especially in individuals at higher risk.

Overall, ECG plays a pivotal role in the comprehensive evaluation and management of ACM patients, providing valuable prognostic information that guides clinical decision making, improves risk stratification, and ultimately enhances patient outcomes. The recognition of these nuanced ECG manifestations has become imperative for clinicians as it guides them in the formulation of tailored therapeutic strategies that address both the present symptoms and potential future risks [1].

This discussion seeks to underscore the dual role of ECG in ACM—unveiling diagnostic clues and providing essential prognostic insights. In doing so, clinicians can intervene more effectively, harnessing the prognostic value of ECG to steer patients toward tailored treatments and, ultimately, improved outcomes.

## 2. Methodological Considerations

To summarize the current evidence related to the prognostic ECG signs in ACM, a systematic review was performed. The review was performed in accordance with the PRISMA (Preferred Reporting Items for Systematic reviews and Meta-Analyses) guidelines [2]. The terms searched were: “((ECG) OR (EKG) OR (electrocardiogram) OR (electrocardiographic)) AND ((sign) OR (predictor) OR (marker)) AND ((arrhythmogenic cardiomyopathy) OR (ARVC) OR (ARVD) OR (right ventricular dysplasia) OR (dysplasia))”. The databases analyzed were PubMed, BioMed Central, and Cochrane Library.

**Figure 1:**
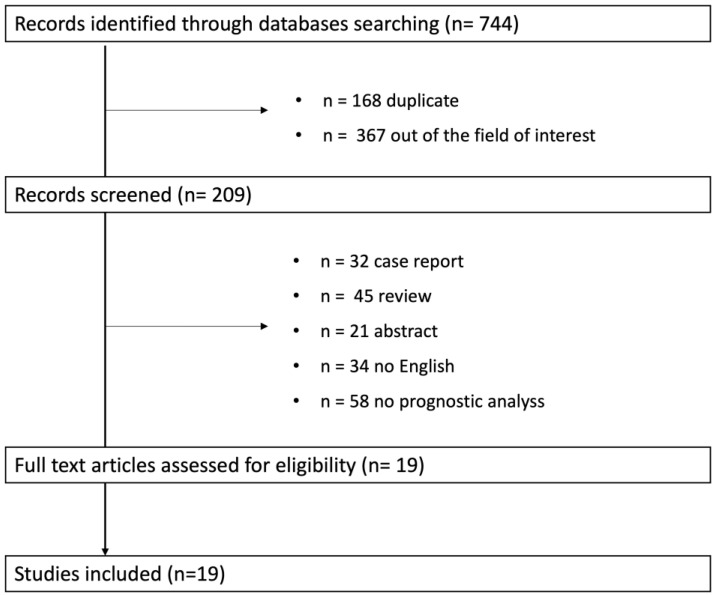
Study flow.

Only papers published in English in or before September 2023 in peer-reviewed journals were selected. The inclusion criteria of the studies were: (1) observational or clinical trials involving patients with ACM. Overall, 744 studies were selected (Figure 1). After a first evaluation, 535 records were screened; of these, 161 were excluded for different reasons (Figure 1).

Finally, 19 studies were included in the systematic review. The quality of the included studies was tested using pre-specified electronic forms of MINORS criteria [3]. The minimum score obtained was 10, and the maximum was 20. No studies were excluded on the basis of quality assessment. In all the studies, ACM was defined as recommended: a definite diagnosis of right ventricular arrhythmogenic cardiomyopathy was fulfilled by 2 major or 1 major and 2 minor criteria or 4 minor criteria from different categories (imaging, histomorphometry, genetics, ECG, and arrhythmic features); the left ventricular involvement was described according to left ventricular dysfunction, fibrofatty infiltration, and ECG signs [1].

## 3. Results

We presented the results of the systematic review, answering questions related to the prognostic role of ECG signs in ACM. As summarized in Figure 1, 19 studies were included. Prospective and retrospective single- and multicenter studies conducted between 1999 and 2023 were analyzed. The main endpoints were sustained ventricular tachycardia, appropriate ICD discharge, and SCD. The ECG signs with significant prognostic values included: TWI, QRS fragmentation, low QRS voltage, epsilon wave, QRS dispersion, repolarization dispersion, terminal activation duration (Table 1).

## 4. T-Wave Inversion

**Figure 2:**
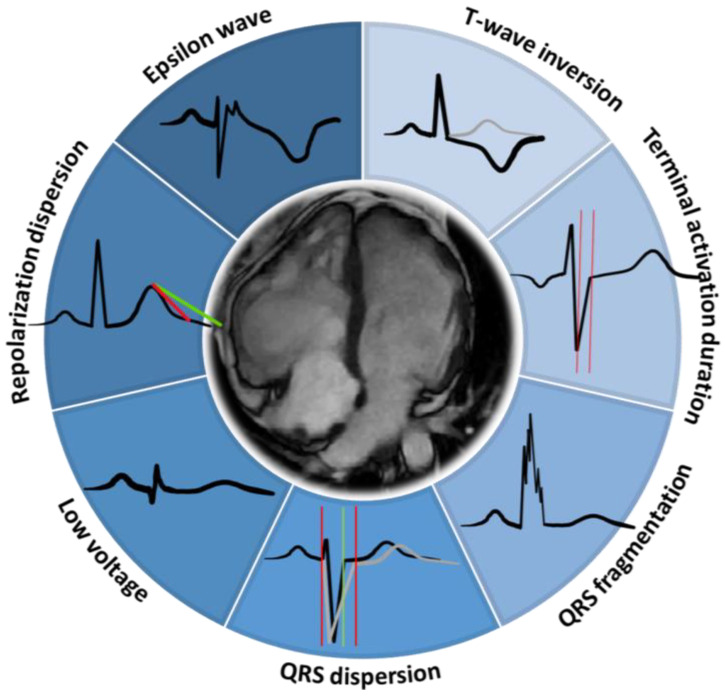
Graphical abstract summarizing ECG signs with prognostic value in ACM.

T-waves are considered inverted if the negative component has an amplitude ≥ 1 mV (1 mm). T-wave inversion (TWI) in V1, V2, and V3, and beyond in otherwise healthy individuals aged 14 years or older, is observed in only 4% of healthy women and 1% of men. Therefore, it is reasonably specific in this population and is considered a major diagnostic abnormality in ACM according to the revised 2010 task force criteria (Figure 2) [1].

Nava et al. found a direct correlation between the extent of T-wave negativity in the precordial leads and right ventricular enlargement (r = 0.89; *p* =< 0.01): T changes could be caused by the backwards dislocation of the left ventricle, which is secondary to RV dilatation, asynchronous RV repolarization, or intraparietal RV conduction defects. So, TWI is an expression of the fibrofatty replacement that represents the arrhythmic substrate that is typical of ACM [22]. In fact, the presence of TWI suggests underlying structural and electrical myocardial alterations, including fibrosis and ion channel dysfunction, which contribute to the arrhythmic substrate [22]. An interesting observation regarding the association between TWI and the arrhythmic substrate emerges from a study by Zorzi et al.; this study demonstrated that the extent of negative T-waves across a 12-lead ECG allows the noninvasive estimation of the amount of right ventricular electroanatomic scar detected by endocardial voltage mapping as well as the prediction of the right ventricular electroanatomic scar-related arrhythmic risk [4]. Similar findings were reported in a retrospective study of 64 patients with ACM by Kalantarian et al.; the study showed that inferior TWIs were predictors of RV enlargement and dysfunction and the risk of any ventricular tachycardia or adverse cardiac outcome [5]. Furthermore, TWI, especially in the lateral precordial leads (V5–V6), represents a sign of left ventricular involvement reflecting myocardial repolarization abnormalities in the affected regions [5].

The prognostic value of the major criterion TWI has been described in a longitudinal cohort study by Bhonsale et al., which showed that an increase in the number of inverted precordial T-waves was associated with an increase in the proportion of subjects experiencing an arrhythmic event. Patients with ≥3 precordial inverted T-waves demonstrated a significantly increased arrhythmic risk compared with participants with T-wave inversion on only one or two right precordial leads. Arrhythmic events among family members occurred only in patients with ≥3 T inversions. An incremental risk pattern with an increasing number of precordial T inversions was also seen for the occurrence of VT storms, appropriate ICD interventions, and non-sustained ventricular tachycardia [6].

Saguner et al. confirm that an incremental relationship between precordial TWI and MACE (cardiac death, heart transplantation, survived sudden cardiac death, ventricular fibrillation, sustained ventricular tachycardia, or arrhythmic syncope) exists, with >3 inverted precordial T-waves conferring an almost twofold increased hazard compared with ≤3 precordial TWIs. An important and novel finding is the independent prognostic value of inferior lead TWIs, which have been linked to left ventricle involvement, which may explain their predictive utility. In multivariable analysis, inferior lead TWIs remained an independent predictor of a major adverse cardiac event (MACE). These reliable and easily recognized abnormalities seem to constitute key elements of arrhythmic risk in patients with ACM, probably because they are associated with severe right ventricle disease as well as left ventricle involvement [7].

Link et al. enrolled 137 patients with ACM to identify the predictors, characteristics, and treatment of ventricular arrhythmias. Of the 137 enrolled patients, 108 received ICDs. Among the patients who underwent ICD implantation, 48 had ventricular arrhythmias treated by the ICD during follow-up. In a multivariate analysis, the only two predictors of ICD treatment for ventricular arrhythmias were pre-implantation sustained monomorphic ventricular tachycardia or sustained polymorphic ventricular fibrillation (*p* = 0.0029) and inferior T-wave inversions (*p* = 0.0159). There were no sudden deaths in the cohort with ICDs or in the cohort who did not receive ICDs. In particular, 22 individuals had the occurrence of rapid, sustained monomorphic ventricular tachycardia (≥240 beats/min) or sustained polymorphic ventricular fibrillation after ICD implantation. In a multivariate analysis, only younger age at the time of ICD implantation was predictive of life-threatening ventricular arrhythmias (*p* = 0.032) [8].

Orgeron et al. in a paper published in 2017, revealed that patients with ACM who undergo ICD implantation for primary or secondary prevention have a remarkably high incidence of appropriate ICD therapies both for any sustained ventricular arrhythmia (22% annually) and for VF/VFL (3.6% annually). Among the clinical variables that were identified as predictors of any appropriate ICD therapy were ≥3 T-wave inversions in ECG, a history of sustained ventricular tachycardia at presentation, inducibility at EPS, male sex, and a PVC count ≥ 1000/24 h [9].

As demonstrated by Laren et al., TWIs are useful for the identification of arrhythmic events even in early ACM [10]. According to these data, the number of leads with TWIs became a predictor in the ACM risk score validated in 2019 by Cadrin-Tourigny et al. [10]. An even more recent study by Cadrin-Tourigny et al. specifically investigated the predictive factors of life-threatening ventricular arrhythmias (LTVAs) to serve as a closer surrogate marker for sudden cardiac death risk. The latter study highlighted the fact that younger age, male sex, PVC count, and number of leads with T-wave inversion were predictive of LTVA [11].

## 5. QRS Fragmentation

QRS fragmentation is defined as the presence of additional deflections/notches at the beginning of the QRS, on top of the R-wave, or in the nadir of the S-wave. Previous studies have suggested a link between QRS fragmentation and myocardial scar, which potentially indicates a poorer prognosis in patients with coronary artery disease [23]. This finding also suggests that QRS fragmentation may reflect myocardial replacement in ACM. Additionally, fragmented QRS complexes may be observed in ACM that also involves the left ventricle, which is suggestive of myocardial fibrosis and conduction disturbances [24]. Notably, QRS fragmentation exhibits less inter-observer variability compared to other depolarization abnormalities like the epsilon wave, although it is not included in the revised task force criteria (TFC) of 2010 [1]. The Roudijk group compared ACM patients and both symptomatic and asymptomatic pathogenic variant carriers with a control group and found that QRS fragmentation was more frequent in both disease groups (including those with a definite diagnosis and those with only gene mutation) [24]. Additionally, they highlighted the utility of this abnormality in diagnosing early-stage disease [24]. Peters et al. retrospectively analyzed 305 ACM patients, of whom 83% had QRS fragmentation; they found that QRS fragmentation within the S-wave of leads V1 and V3 (68% of the study population) was significantly associated with arrhythmic events, including ventricular tachycardia, primary ventricular fibrillation, sudden cardiac death, and recurrent ICD discharges [12]. In a group of 111 patients, that included possible, borderline, and definite ACM diagnosis according to the 2010 revised TFC, QRS fragmentation constituted an independent predictor of major adverse cardiovascular events (MACE), with a hazard ratio (HR) of 2.65 (1.1 to 6.34), *p* < 0.029), retaining its significance even in a subgroup of patients with low arrhythmic risk, i.e., those without previous SCD, VF, and/or sustained VT [13].

## 6. Low Voltage QRS

Low QRS voltage occurs in several cardiomyopathies and pericardial disease. Its specificity for ACM is low [25]. Recently, the Padua criteria included low QRS voltage in limb leads as an ECG minor criterion of diagnosis [26]. The presence of low QRS voltage in limb leads has been reported to be specific for left ventricular involvement in ACM. The mechanism seems to be related to the decrease in left ventricular mass. Furthermore, this sign correlates with a high number of left ventricular segments affected by LGE [26]. Although its diagnostic role is not already validated, there are some data supporting its prognostic value. Effectively, the low QRS voltage is an ECG marker of a high degree of fibrofatty infiltration or fibrosis, which also involves the left ventricle, and of a more severe clinical profile as well. A recent cohort study by Olivetti et al. suggests that the presence and the extension of low QRS voltage can be an independent risk predictor associated with heart failure death and heart transplantation in ACM patients, regardless of the arrhythmic risk [14].

## 7. Epsilon Wave

The epsilon wave is defined as a low-amplitude deflection following the QRS complex in the right precordial leads. The epsilon wave primarily arises from delayed right ventricular activation, which is attributable to impaired electrical propagation through isolated myocytes amidst the fibrofatty tissue substitution [15]. Furthermore, the epsilon wave may only manifest during exercise testing, suggesting its association with the early stage of disease development [27,28]. Regarding its prognostic significance, the literature remains inconclusive. In a population of 68 ACM patients, Gallo et al. reported a link between epsilon waves and an increased risk of the composite endpoint of sudden cardiac death, heart failure-related mortality, and heart transplantation, with an odds ratio (OR) of 20.9 (1.8–239.8, *p* = 0.015), suggesting a potential role in disease progression [15]. In contrast, Turrini et al. found no significant difference in epsilon wave prevalence among patients with ACM across varying risk profiles [29]. Wu et al. observed a trend towards poorer prognosis in patients with epsilon waves, but without high statistical significance [30]. In a population of 215 patients harboring ACM-related mutations, Bhonsale et al. demonstrated a correlation between the presence of either major depolarization abnormalities (e.g., epsilon wave and QRS duration exceeding 110 ms in the right precordial leads) or minor depolarization abnormalities (e.g., terminal activation duration and late potentials in a signal-averaged ECG) and an increased risk of adverse outcomes. However, patients without any depolarization abnormalities also had low event-free survival, suggesting that this parameter was not a good discriminator on its own. On the other hand, the integration of information from both depolarization and repolarization abnormalities appeared to be valuable in stratifying patients based on their risk of life-threatening arrhythmias. They identify high-risk patients as those with ≥3 precordial inverted T-waves, intermediate-risk patients as those with an isolated depolarization abnormality or TWI in V1–2 and depolarization abnormalities, and low-risk patients as those with less than two TWIs and no repolarization abnormalities [6]. In an event-free population of 115 ACM patients, the presence of depolarization abnormalities, i.e., the epsilon wave, was not associated with an increased risk of life-threatening ventricular arrhythmias [31].

## 8. QRS Dispersion

QRS dispersion, defined as the difference between the longest and shortest QRS duration in any lead, reflects regional variations in depolarization timing. It has been associated with both right ventricular (RV) diameter and volume, as assessed by cardiac magnetic resonance (CMR), and also with a late gadolinium enhancement (LGE) detection rate, which may have prognostic importance in assessing the risk of sudden death [32]. A study by Turrini et al. compared 4 groups: 20 sudden death victims, 20 ACM patients with history of ventricular arrhythmias, 20 ACM patients with less than 3 premature ventricular beats (PVBs), and 20 controls. The analysis demonstrated that a QRS dispersion ≥ 40 ms was an independent predictor of sudden cardiac death, with a sensitivity and specificity of 90% and 77%, respectively [16]. Also, Peter S et al. observed a correlation between QRS dispersion, defined in this case as ≥50 ms, and recurrent episodes of ventricular arrhythmias [17]. However, both studies lacked long-term follow up data. Hsieh’s group analyzed the QRS dispersion as the angular difference between the reconstruction vectors obtained from the QRS loop decomposition, based on a principal component analysis, and created the variable defined as interlead QRS dispersion (IQRSD). They evaluated IQRSD in four groups of patients: those with ACM who had undergone epicardial ablation, those with ACM with ventricular tachycardia (VT), those with an idiopathic right ventricular outflow tract (RVOT) VT, and a control group. IQRSD was effectively able to distinguish the ACM patients from the controls and could identify patients with more advanced disease who required epicardial ablation intervention [18].

## 9. Repolarization Dispersion

Repolarization abnormalities are some of the significant contributors to life threatening arrhythmias and mortality, and they are frequently observed in electrocardiograms of patients with ACM. Specifically, repolarization dispersion, which arises from uneven refractory periods and activation times, reflects the underlying pathological substrate of fibrofatty myocardial replacement. Several ECG parameters have been proposed to identify repolarization dispersion, with the most common being the interlead variability in the QT interval duration (classified as abnormal when greater than 65 ms). Regarding its prognostic value, the literature is inconclusive. On one hand, Turrini et al. reported significatively higher QT dispersion in patients who had experienced sudden death compared with living patients with various arrhythmic profiles (defined based on a history of sustained ventricular tachycardia); they also confirmed the 65 ms cut-off as a valuable tool for refining risk stratification [16]. On the other hand, Benn et al. found no correlation between QT dispersion and the severity of symptoms, while still supporting its role in distinguishing ACM patients from healthy controls [19]. Repolarization dispersion was further investigated using other parameters, including the Tp-e interval, cTp-e interval, Tp-e/QT ratio, and Tp-e/QTc ratio. They were observed to be prolonged in ACM patients compared with healthy subjects. In multivariate analyses, the c Tp-e interval was also significantly associated with all-cause mortality, with an odds ratio (OR) of 1.166 (1.017–1.336, *p* = 0.027) [20].

## 10. Terminal Activation Duration

Terminal activation duration (TAD) is measured from the S-nadir to the end of all depolarization deflections, and it is considered prolonged when it exceeds 55 ms in any of the leads V1–3. In the absence of a right bundle branch block, it is considered to be a minor diagnostic criterion for ACM. TAD has been also described as an extension of the “S wave upstroke”, and it could be considered a marker of delayed right ventricular activation, which has been suggested to play a role in the pathogenesis of ventricular tachycardia (VT) in ACM [21].

## 11. Conclusions and Future Directions

**Figure 3:**
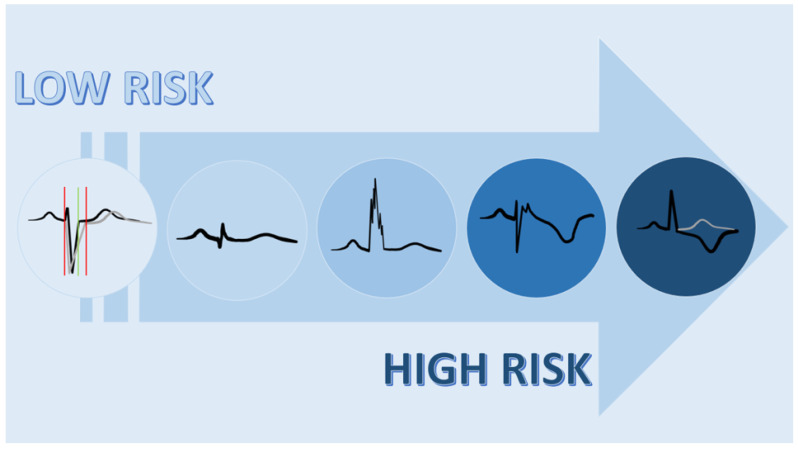
ECG signs with increasing prognostic value in ACM.

The ECG findings regarding ACM showed a validated diagnostic role, a good correlation with imaging features, and a potential prognostic value (Figure 2). In fact, there are some signs such as TWI that showed an important correlation with prognosis [22]. Figure 3 shows an ACM ECG sign according to increasing prognostic value. Combined ECG signs in ACM can carry significant prognostic implications, aiding in risk stratification and management decisions. Patients presenting with multiple ECG abnormalities are often at higher risk for adverse cardiac events, including ventricular arrhythmias and sudden cardiac death because of a more extensive myocardial involvement [26].

T-wave inversion in the precordial leads combined with other ECG abnormalities, such as QRS prolongation or fragmented QRS complexes, suggests more extensive myocardial fibrosis and electrical instability, which correlate with an increased risk of arrhythmias. Additionally, low voltage QRS complexes may indicate advanced myocardial involvement and fibrosis, further elevating the risk of adverse outcomes [26].

**Figure 4:**
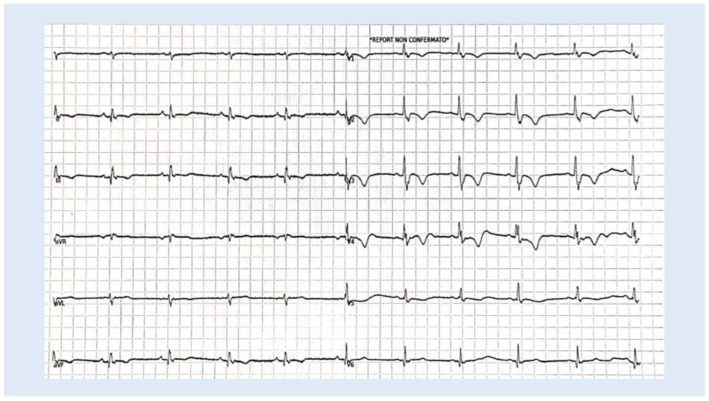
An ECG of a 42-year-old man summarizing ACM prognostic signs.

Clinicians use these combined ECG signs as part of a comprehensive risk assessment in ACM patients. Those with multiple ECG abnormalities may require closer monitoring and aggressive management of arrhythmic risk factors. Figure 4 shows an ECG of a 42-year-old man with biventricular ACM and combined ECG signs. However, some challenges and limitations have to be considered, such as the phenotypic variability observed among patients. The disease manifests differently in individuals, making it crucial to identify the specific ECG patterns associated with distinct clinical outcomes.

Genetic factors play a significant role in ACM and understanding the genotype–phenotype correlation is crucial for personalized risk stratification [1]. Future research should focus on integrating genetic data with ECG findings to enhance prognostic accuracy.

Future studies should focus on combining ECG data with advanced imaging modalities, such as cardiac magnetic resonance imaging (CMR); it may provide a comprehensive understanding of ACM pathophysiology. The integration of multimodal data could refine risk stratification and guide therapeutic interventions.

Additionally, longitudinal studies with extended follow-up periods are imperative to capture the dynamic changes in ECG patterns and their correlation with clinical outcomes. The ECG changes noted during the follow-up of ACM patients have generally been perceived by investigators as evidence of disease progression, possibly reflecting areas of new myocardial involvement or progressive adverse remodeling. It may be hypothesized that dynamic ECG changes in ACM are an early disease expression related to gap junction and desmosome remodeling, in the absence of detectable clinical structural or functional alterations or the typical histological changes in the fibrofatty replacement. In contrast, when overt disease is present, ECG abnormalities may be stable or progressive, presumably because significant structural changes have occurred.

The main data that are currently available are derived from a retrospective analysis; prospective cohorts will contribute valuable insights into the progression of ACM and aid in refining prognostic models.

The prognostic role of ECG in ACM is an evolving field with exciting prospects. As our understanding of the disease deepens and technology advances, integrating multiple modalities and refining predictive models will undoubtedly contribute to improved patient outcomes. Collaborative efforts among clinicians, researchers, and technology developers are essential to unravel the complexities of ACM and to pave the way for more effective diagnostic and therapeutic strategies.

## Figures and Tables

**Table 1:** ECG signs and their prognostic value.

	Main Characteristics	Prognostic Value	
T-wave inversion	Single-center retrospective study, 49 pts, year of publication 2013 [4]	Right ventricular electroanatomic scar-related arrhythmic risk	*p* = 0.03
	Single-center retrospective study, 405 ECG, year of publication 2021 [5]	Any ventricular tachycardia or adverse cardiac outcome	*p* = 0.04
	Single-center retrospective study, 215 pts, year of publication 2013 [6]	Arrhythmic events including VT storms, appropriate ICD interventions and NSVT	*p* = 0.035
	Single-center retrospective study, 111 pts, year of publication 2015 [7]	MACE	*p* = 0.020
	Single-center prospective study, 137 pts, year of publication 2015 [8]	ICD treatment of VT	*p* < 0.001
	Single-center prospective study, 312 pts, year of publication 2017 [9]	Appropriate ICD intervention	*p* = 0.018
	Single-center retrospective study, 162 pts, year of publication 2017 [10]	Arrhythmic events even in early ACM	*p* < 0.001
	Multicenter retrospective study, 864 pts, year of publication 2021 [11]	LTVA	*p* = 0.024
QRS fragmentation	Multicenter retrospective study, 335 pts, year of publication 2012 [12]	Arrhythmic events including VT, VF, SCD, and recurrent ICD discharges	*p* < 0.001
	Multicenter retrospective study, 360 pts, year of publication 2008 [13]	MACE	*p* < 0.029
LQRSV	Single-center prospective study, 111 pts, year of publication 2023 [14]	Heart failure death and heart transplantation	*p* = 0.010
Epsilon wave	Single-center prospective study, 68 pts, year of publication 2016 [15]	Composite endpoint of SCD, heart failure-related mortality, and heart transplantation	*p* = 0.015
QRS dispersion	Single-center retrospective study, 40 pts, year of publication 2001 [16]	SCD	*p* < 0.0001
	Single-center retrospective study, 121 pts, year of publication 1999 [17]	Recurrence of ventricular arrhythmias	*p* < 0.01
	Single-center retrospective study, 71 pts, year of publication 2017 [18]	More advanced disease requiring epicardial ablation	*p* < 0.001
	Single-center retrospective study, 25 pts, year of publication 1999 [19]	SCD, VT	*p* < 0.05
Repolarization dispersion	Single-center retrospective study, 40 pts, year of publication 2001 [16]	SCD	*p* < 0.0001
	Single-center prospective study, 105 pts, year of publication 2019 [20]	c Tp-e interval > all-cause mortality	*p* = 0.027
TAD	Single-center retrospective study, 57 pts, year of publication 2009 [21]	Delayed right ventricular activation, playing a role in ventricular tachycardia	*p* < 0.05

## Data Availability

Not applicable.
